# Exploring the genetic architecture of protein secretion in *Komagataella phaffii* (*Pichia pastoris*) for biotechnology applications

**DOI:** 10.1371/journal.pbio.3001911

**Published:** 2022-12-16

**Authors:** Chantle Reiko Swichkow, Joshua S. Bloom, Leonid Kruglyak

**Affiliations:** 1 Department of Human Genetics, University of California, Los Angeles, Los Angeles, California, United States of America; 2 Department of Biological Chemistry, University of California, Los Angeles, Los Angeles, California, United States of America; 3 Howard Hughes Medical Institute, University of California, Los Angeles, Los Angeles, California, United States of America

## Abstract

Improvements to the production of proteins in industrial yeast species have largely relied on engineering in a single genetic background. This Primer explores a new approach described in PLOS Biology which leverages natural genetic variation to identify genes and variants with the potential to improve protein yield.

## Teaser

Improvements to the production of proteins in industrial yeast species have largely relied on generating variation in a single genetic background. A new study in *PLOS Biology* leverages natural genetic variation to identify genes and variants with the potential to improve protein yield.

*Komagataella phaffii* (previously known as *Pichia pastoris*) is an industrial yeast widely used in protein manufacturing for biotechnological and pharmaceutical applications. Compared to the more familiar baker’s yeast *Saccharomyces cerevisiae*, *K*. *phaffii* exhibits higher heterologous protein expression, grows to higher cell densities, can use methanol as a sole carbon source, and is more suitable for posttranslational modification of proteins through folding, glycosylation, and disulfide bonding [1]. These advantages, coupled with the fact that most experimental techniques developed in *S*. *cerevisiae* readily carry over to *K*. *phaffii*, make this species an ideal eukaryotic expression system [2]. Recently, powerful bioengineering approaches have been developed to improve the yield of expressed and secreted proteins in *K*. *phaffii* [3].

**Fig 1:**
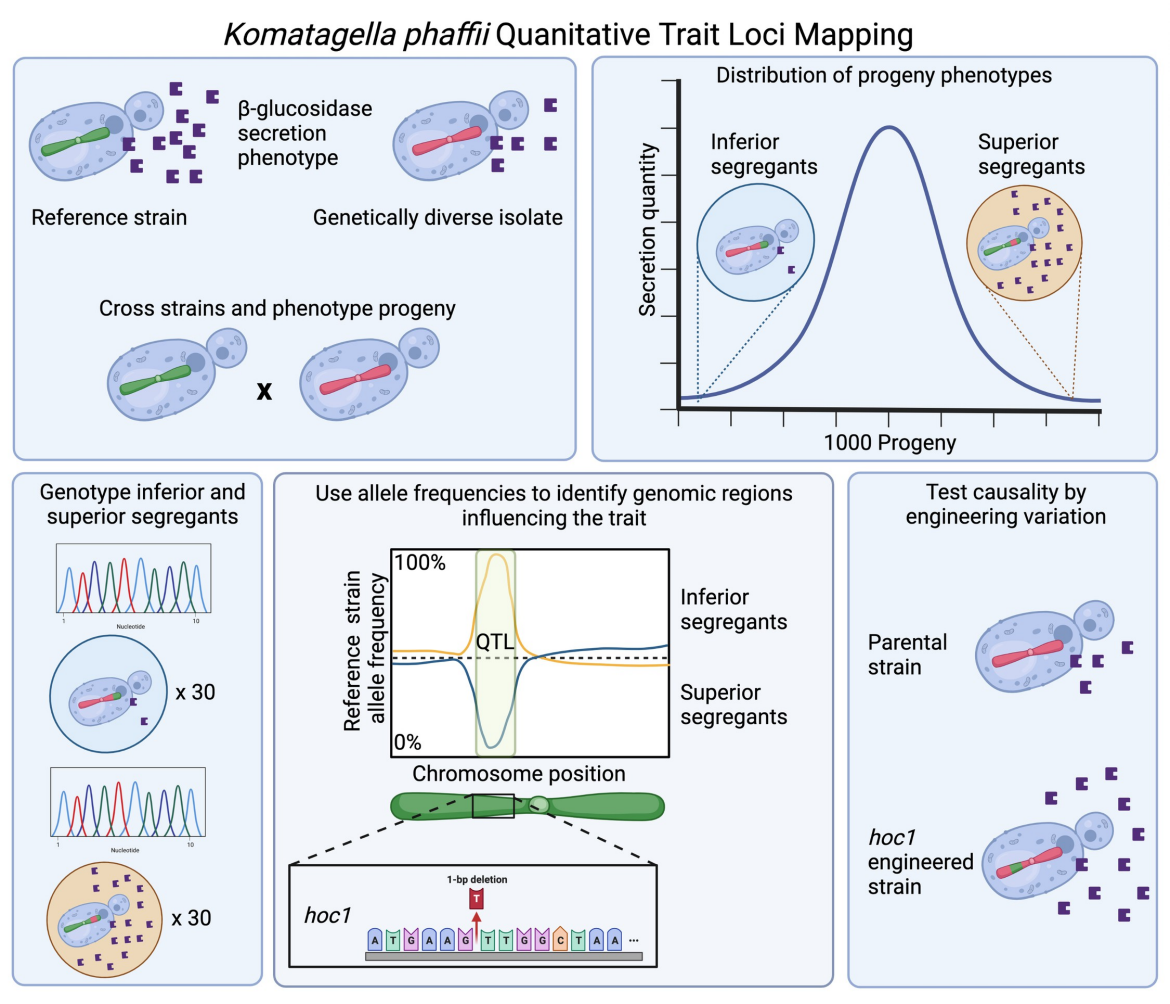
Schematic representation for mapping protein secretion quantitative trait loci. The reference strain of *K*. *phaffii* and a genetically diverse isolate differ in the secretion quantity of a heterologous protein, β-glucosidase. The authors crossed the 2 strains and isolated 1,000 recombinant haploid progeny (segregants). They then measured β-glucosidase secretion in each of the 1,000 segregants and selected those segregants with extreme phenotypes for whole-genome sequencing. This enabled the authors to compute the frequency of each parental allele in inferior and superior segregants at variant sites across the genome. They identified QTL as regions at which allele frequencies diverged between inferior and superior segregants. Sequence variants in candidate genes within the QTL were engineered into the parental strain to test whether they caused differences in β-glucosidase secretion. Figure created with BioRender.com.

In a new study published in *PLOS Biology*, Offei and colleagues use quantitative trait loci (QTL) mapping to investigate how natural genetic variation can affect the yield of heterologous protein secretion in *K*. *phaffii* [4] (Fig 1). Depending on the extent of genetic diversity within a species, any 2 yeast strains may differ by tens of thousands of differences in their genome sequence [5]. The goal of QTL mapping is to identify which of these genetic variants cause a substantial difference in a trait of interest. QTL mapping leverages segregation and recombination during meiosis to randomize the inheritance of parental alleles in the progeny of a cross between genetically distinct individuals. Typically, each progeny strain is then phenotyped for the trait(s) of interest and genotyped at many positions across the genome to track its unique patterns of inheritance of parental alleles. QTL are identified as regions of the genome at which trait values across many progeny are significantly correlated with the inheritance of parental alleles (that is, inheriting one parental allele is associated with a higher trait value than inheriting the other). In the current study [4], this approach identified QTL affecting protein secretion that could inform process refinement and improve industrial yield.

Offei and colleagues first characterized heterologous protein secretion in 3 strains of *K*. *phaffii*—the reference strain CBS7435 (CBS) and isolates Pp2 and Pp4—by integrating a *Thermoascus aurantiacus* β-glucosidase gene sequence (*bgl1*) with an *S*. *cerevisiae* mating factor secretion signal into each strain. They measured secretion of BGL protein in microtiter plates and observed that CBS secreted twice as much protein as the other 2 isolates. The authors then performed genetic crosses between CBS and each Pp isolate and measured BGL secretion in 1,000 segregants (haploid progeny) from each cross. They selected 30 segregants showing superior secretion and 30 segregants showing inferior secretion from each cross and subjected them to individual whole-genome sequencing. This enabled Offei and colleagues to quantify the frequency of each parental allele at approximately 42,000 variant sites. Sites that are not associated with protein secretion are expected to show equal frequencies of the 2 alleles in each segregant group, while sites linked to variants that alter BGL secretion are expected to show opposing deviations from 50:50 in the superior and inferior pools.

The authors ultimately identified 3 QTLs linked to BGL secretion. One locus (QTL1) showed up in both crosses, with superior secretion driven by the CBS allele. Each cross also identified 1 additional locus (QTL2 and QTL3), with superior alleles coming from Pp2 and Pp4, respectively. The QTL intervals contained multiple genes, and the authors leveraged additional biological information and predictions of variant effects to select 2 genes for experimental follow-up. The authors narrowed QTL1 to a frameshift mutation (*hoc1*) in the *HOC1* gene, which plays a role in cell wall biosynthesis. Disruption of the *HOC1* gene in Pp2 and Pp4 by CRISPR/Cas9 increased BGL secretion 2-fold, while correction of the frameshift in CBS decreased BGL secretion by half. Strains carrying the *hoc1* allele showed increased sensitivity to a cell wall-perturbing agent. The *hoc1* allele likely arose during selection by Phillips Petroleum for increased methanol uptake and greater cell wall permeability [6].

The QTL2 interval on chromosome 1 spanned 18 genes. Offei and colleagues [4] selected *IRA1* as a strong mechanistic candidate gene that carried a non-synonymous mutation in Pp2 relative to the other 2 strains. The authors showed that deletion of the CBS *IRA1* allele in a CBS × Pp2 diploid strain increased BGL secretion, suggesting that the Pp2 *IRA1* allele is recessive and beneficial for protein secretion. However, this allele did not increase secretion of 2 other heterologous proteins, indicating that it may be specific to BGL and not applicable to improving the process more generally.

The study by Offei and colleagues demonstrates the feasibility of carrying out QTL mapping in a nonconventional yeast species to identify genes and variation that influence industrially important phenotypes. To date, QTL mapping in the conventional yeast *S*. *cerevisiae* has been extremely successful. This success stems, at least partly, from the availability of many diverse isolates that vary in many phenotypes of interest. In addition, developing genetic markers for genotyping previously required major effort in non-model organisms [7]. However, advances in next-generation DNA sequencing make it more accessible for communities working in non-model organisms to bootstrap genetic studies for phenotypes of interest and genome engineering via CRISPR streamlines downstream functional analysis of genes and variants. This is likely to be the first of many QTL studies in *K*. *phaffii* as the field discovers, sequences, and engineers additional novel strains with interesting phenotypes [8].
